# Malnutrition in children under five years in a squatter settlement of Karachi: a case-control study

**DOI:** 10.1186/s12889-024-18359-3

**Published:** 2024-03-19

**Authors:** Gati Ara, Bina Fawad, Shumaila Shabbir

**Affiliations:** 1 Dow University of Health Sciences, Karachi, Pakistan; 2 Ziauddin University, Karachi, Pakistan; 3 FRPMC, Air University, Karachi, Pakistan

**Keywords:** Children under five years, Malnutrition, Risk factors, Case- control, Squatter settlement, Karachi

## Abstract

**Background:**

Multidimensional factors such as socioeconomic or environmental factors influence malnutrition. Several studies have strongly linked malnutrition to poverty. Some international studies point to the worse nutritional status of urban slum children than rural children. Limited data is available regarding the nutritional status of slum children in Karachi. This study aimed to determine characteristics of malnourished children in an urban squatter settlement in Karachi, Pakistan.

**Methods:**

A case- control study was carried out at the primary healthcare center of a squatter settlement in Karachi, Pakistan. All children under five years of age visiting the primary healthcare center were recruited consecutively. Cases were defined as children with z scores < -2 SD of WHO reference measurements of WFA, HFA, WFH and OFC. The controls were similar in terms of age group but had z scores between − 2SD and + 2SD. A self- structured risk factor questionnaire that included information about sociodemographic, economic and environmental factors as well as child- related characteristics was researcher administered via face-to-face interviews with the mothers of children. Univariate and multivariate logistic regression analyses were conducted. Crude and adjusted odds ratios were calculated with 95% confidence interval.

**Results:**

A total of 280 participants including 140 cases and 140 controls participated in the study. A larger proportion of the sample originated from individuals with low household income. After adjusting for the confounders, childhood malnutrition was significantly associated with a low education level of father (aOR 4.86, 95% CI 2.23–10.60), a monthly income less than 25,000 PKR (89 USD) per month (aOR 7.13, 95% CI 1.67–30.54), pour pit latrine type of toilet (aOR 4.41, 95% CI 2.67–7.3), less than six months of exclusive breast feeding (aOR 3.578, CI 1.58–8.08), inappropriate weaning age (aOR 3.71, 95% CI 1.53-9).

**Conclusion:**

Malnutrition in children under five years of age in the community is associated with low family income, low paternal education, poor toilet facilities, lack of exclusive breastfeeding and inappropriate weaning age. The implementation of poverty reduction programs, sanitation provision at affordable rates, community-based breast feeding and weaning education intervention are urgently required to efficiently improve children’s nutritional status.

## Background

Malnutrition refers to the deficiencies or immoderations in a person’s intake of energy and/or nutrients. Children are the most severely affected, and young children are significantly more vulnerable. The World Health Organization has identified malnutrition as the single most dangerous threat to global public health [1]. The maximum burden of malnourished children is in Asia, with almost 70% of malnourished children dwelling there [2]. In comparison to other developing countries, Pakistan has one of the highest burdens of child malnutrition [3]. As reported by the National Nutrition Survey (NNS) 2018, 40.2% children below the age of five years are stunted, 29% are underweight and 17.7% suffer from wasting in Pakistan [4]. Baluchistan and Sindh are two of Pakistan’s provinces with the highest prevalence of malnutrition and poverty [5]. In the province of Sindh, 42% of children under five years are underweight, and approximately half are stunted (48%) [6]. 

Multidimensional factors such as socioeconomic or environmental factors influence development of malnutrition. Several studies have strongly linked malnutrition to poverty [7]. Poverty is connected to insufficient food, poor sanitation, maternal depression, stress and low maternal education, as well as scarce stimulation at home [8]. All these factors detrimentally affect child development. According to the World Bank, the poverty ratio in Pakistan in 2020-21 is 39.3% based on a lower- middle- income poverty rate of $3.2 per day [9]. Several past studies have explored various risk factors for childhood malnutrition, such as education of mothers/parents, occupation of the head of the family, family income and size, residence location and land availability; these factors have all been documented to influence nutritional status of children [10]. 

In Pakistan, malnutrition studies mostly target rural populations. No recent studies have specifically targeted the under-five children of urban slums in Karachi. Studies that have targeted slums populations are either very old, related to older children or specific micronutrient deficiencies. One malnutrition survey was conducted in Karachi’s Mahmoudabad slum area in 1989 [11]. The latest national estimates are available from the National Nutrition survey (NNS) 2018. The NNS segregates population on the basis of province, city, district and urban/ rural residence, but it does not specify smaller urban squatter settlements. The findings of NNS 2018 are representative of the district level [4]. In the city of Karachi, the disparity within districts can be very high, with high- level housing areas attached to a squatter settlement within the same districts [12]. Some international studies point to a worse nutritional status of urban slum children compared to that of national, urban and rural children. Gosh S et al. stated that children residing in urban slums have the worst nutritional status among all urban groups and are even below the rural level [13]. The urban slums consist of marginalized communities and are often ignored. Several risk factors for malnutrition have been identified and may seem understood, but which risk factors should be prioritized targets for future nutritional intervention in urban squatter communities needs to be documented. The objective of this study was to measure the risk factors for malnutrition in children under five years of age in a squatter settlement in Karachi, Pakistan. This study highlighted the risk factors of malnutrition for under five children of urban slums and could help in further prioritizing selective risk factors for sustainable, cost-effective interventions for controlling and preventing malnutrition in urban squatters.

## Methods

### Study design and setting

This case-control study was conducted from December 2021 to December 2022 in the Gulshan- e- Sikenderabad area, an urban squatter settlement in the Keamari area of Karachi, Pakistan. The area is divided into five blocks and the estimated population was 35,000 approx. in 2010 [14]. The current (undocumented) population estimates by community health workers range from approximately 75,000 to 100,000. Most of the people in the community belong to lower socioeconomic status. There is no government healthcare facility running in the area. There are few dispensaries running in the area by quacks. Some private and nongovernmental organizations are operating in the vicinity. Most of the population consists of Pashtoon in-migrants from KPK or immigrants from Afghanistan with a small proportion of in-migrants from other parts of Pakistan, such as southern Punjab (Saraiki). The population consists of low-income, large, poorly spaced families with birth rate higher than the city average [14]. 

### Study population and sampling

All children under five years of age visiting the primary healthcare center in Sikenderabad, and residents of the settlement were consecutively enrolled after informed written consent from parents. Screening for malnutrition was done using anthropometric measurements. Malnourished children were identified and selected as cases. The cases were defined as children with z scores less than − 2 SD from the median of the WHO reference for anthropometric measurements of Weight-for-age (WFA), Height-for-age (HFA), Weight-for-height (WFH) and head circumference. All children regardless of the type of malnutrition; underweight, stunting, wasting or combination of all were eligible. The controls were children who visited the same healthcare center and belonged to the same age group, however, had z scores between − 2SD and + 2SD measurements of WFA, HFA, WFH and head circumference.

All children under the age of five years and residing in the Sikenderabad area were eligible for inclusion. The Children who were diagnosed cases of special needs, had an ongoing serious or chronic illness, had physical challenges, diagnosed mental illness or mental retardation or were Pre- term babies (less than 37 weeks gestation) were excluded from the study. Children with severe acute illnesses or severe dehydration, hypothermia, hypoglycemia, or hyperpyrexia were also excluded.

### Sample size determination

The sample size was calculated from openepi.com, Version 3, open-source calculator for case-control study. Since the local prevalence of other risk factors was lacking, poverty was the major risk factor and was taken as exposure. In Pakistan, the probability of exposure to poverty (< 3.2 USD) among controls is 0.393 [9]. The odds ratio for a poor household with malnourished children as compared to non-poor house hold is 2.15 [15]. The sample size was calculated at α = 0.05 with 80% power of study and a 1:1 ratio of cases to controls. The hypothetical proportion of controls with exposure was 39.3% and the hypothetical proportion of cases with exposure at the OR of 2.15 was 58.19. The sample size with continuity correction was thus calculated to be 120 cases and 120 controls making a total of 240. Data was collected from a total of 280 participants (140 cases and 140 controls) considering a non- response rate ≥ 16%. A total of 280 respondents were finalized for the study. Estimated 140 cases and 140 controls were included in the study.

### Data collection and measurement of variables

After informed written consent was obtained from the parents, the anthropometric measurements were taken by the principal investigator and community health worker following WHO protocol for measurement of height, weight, and head circumference for the Indicators of Malnutrition: Underweight (low Weight-for-age), Stunting (low Height-for-age), Wasting (low Weight-for-height), and low Occipitofrontal circumference. The Mid Upper Arm Circumference (MUAC) was not measured. The nutritional status was examined, and the children were classified and enrolled based on the selection criteria. The children were not assessed for any co-morbidities other than those that warranted exclusion. The risk factors were assessed through self -structured questionnaire that included questions regarding sociodemographic, economic and environmental characteristics; feeding practices; and the frequency of common illnesses. The research investigator collected data on risk factors via questionnaire administered via face- to- face interviews with mothers of children in Urdu language. One child corresponded to only one household. Only mothers of children were interviewed to minimize recall bias. When the mothers were not living with the participants or were deceased, the primary guardian was interviewed.

The outcome variable was malnutrition, and the key risk factors were low income, history of migration, mother’s lack of education, father’s lack of education, nuclear family system, two or less rooms, inaccessibility to proper flush toilet facilities, large number of family members, high number of children, more than one child under the age of five years, home delivery, low birth weight, delivery by a relative or unskilled attendant. In-exclusive breast feeding of infants under 6 months, lack of two years breast feeding, early or late weaning, frequency of recurrent illnesses, history of worm infestation, incomplete EPI immunization.

### Data processing and analysis

All duly filled forms were verified manually, and errors were identified and rectified. Incomplete forms were completed, and verified data were entered into SPSS version 24 for further processing. A backup depository was developed to prevent data loss. Descriptive statistics such as the mean and standard deviation were used for quantitative variables like age, weight, height, head circumference, birth weight, house hold size, number of siblings, number of siblings under two years of age, and house hold income. The percentage and frequency were utilized for qualitative variables such as sex, ethnicity, housing, water source, immunization status, parents’ educational status, parents working status, father’s job type, and father’s profession. Predictors of childhood malnutrition were investigated using logistic regression analysis. A variety of household, maternal and child- related factors were included. First a univariate analysis was conducted to determine the independent effect of each predictor on the outcome. All the predictors were subsequently included in multivariate analysis to investigate their net effect after adjusting for age of child, source of water, presence of cemented or uncemented house, number of people using one toilet and all other factors in the model. Adjusted ORs were calculated to study the predictive power of independent variables in relation to malnutrition. The statistical significance was set at a *p* value < 0.05.

### Ethical approval

Approval for the research was obtained from the Institutional Review Board of Dow University of Health Sciences, Karachi, Pakistan. Reference: IRB-2345/DUHS/Approval/2021/892. Formal permission was obtained from Ziauddin University’s Department of Family Medicine for data collection from the Primary Healthcare Center.

## Results

The total sample size was *n* = 280 with 140 cases and 140 controls. The mean age of the sample was 31.2 ± 18 months. The mean quantitative variables of cases and controls are given in Table 1.

**Table 1:** Description of the study participants:

Factors	Cases (*n* = 140) Mean + std. deviation	Controls (*n* = 140) Mean + std. deviation
Age in months	32.6 ± 16.25	29.6 ± 19.6
Weight in kg	11.3 ± 4.2	11.9 ± 3.9
Height in cm	80 ± 11.6	84.7 ± 17
OFC in cm	48 ± 5.7	47.6 ± 4.5
Mean Birth weight in kg	2.58 ± 1.7	2.59 ± 0.3
Exclusive breast feeding (months)	4.01 ± 2.87	4.7 ± 1.7
Weaning age in months	8.2 ± 3.7	6.65 ± 1.67
Monthly income in PKR:	12,042 ± 6276	16,754 ± 15,326
Number of immediate family members	6.5 ± 1.8	6.1 ± 1.7
Number of total household members	9.2 ± 5	8.8 ± 4
Number of siblings	3.7 ± 1.8	3.2 ± 1.6
Number of siblings < 2 yrs.	0.5571	0.471
Period of residence in Karachi (years)	12.49 ± 10.8	15.78 ± 12.49

The sociodemographic factors, and the associations of various social, economic and environmental factors with malnutrition status are given in Tables 2 and 3, respectively.

**Table 2:** The sociodemographic characteristics of the study population

Sociodemographic characteristics	Cases *n* = 140 (%)	Controls *n* = 140 (%)
**Gender**		
Male *n* = 145	79 (56.4)	66(47.1)
Female *n* = 135	61 (43.57)	74 (52.85)
**Ethnicity**		
Pushtun (from Pakistan)	58 (41.4)	70(50)
Saraiki	30 (21.4)	22(15.71)
Pashtun (from Afghanistan)	24 ((17.1)	20(14.2)
Punjabi	22(15.71)	18(12.8)
Sindhi	5(3.5)	7(5)
Urdu Speaking	1(0.07)	1(0.07)
Other	0	2(1.4)
**Migration**		
Settled in Karachi since < 15years	93(66.4)	80(57.1)
Settled in Karachi since > 15 years	47((33.6)	60(42.8)
**Father’s Educational level**		
No education	48(34.3)	40(28.57)
Primary or less	67(47.8)	50(35.7)
Secondary	25(17.8)	48(34.2)
Graduate/Post graduate	0	2(1.4)
**Mother’s Educational level**		
No education	91(65)	70(50)
Primary or less	43(30.7)	58(41.4)
Secondary	6(4.2)	12(8.5)
Graduate/Post graduate	0	0
**Mother’s working status**		
Housewife	120	123
Working	20	17
**Father’s working status**		
Jobless	4	5
Employed	136	135
**Father’s job type**		
Daily wage	78(55.7)	57(40)
Temporary	52 (37.1)	67(47.8)
Permanent	10(7.1)	17(12.1)
**House hold type**		
Joint/Extended family	53(38)	52(37.1)
Nuclear family	87(62)	88(62.8)
**Monthly Household Income** In PKR ($)		
< 25,000 (<$89)	131(93.6)	115(82.1)
≥ 25,000 (≥$89)	9(6.4)	25(17.8)

### Breastfeeding practices

The mean age of exclusive breastfeeding was 4+/- 2.4 months. Mean age of in-exclusive breastfeeding was 19 +/- 9 months. Only 108 (38.5%) participants (Cases + Controls) were exclusively breastfed for first six months of life. A total of 145 (52%) participants had exclusive breastfeeding for less than six months, and 20 (7%) had exclusive breastfeeding for more than six months. Various other types of products were used during the first six months by the majority of the participants. Total 18% had used formula, 29% had Buffalo or Cow milk, and 2% had goat milk. A total 6.4% infants were given a tea whitener during the first six months of life, out of which 4.7% were cases and 1.7% were controls,

Among the 172 (61.5%) participants using products other than breast milk, feeding bottle was the most common method of giving products and was used by 148 (86%) participants. Only 9 (5%) participants were given non breast milk products via cup and spoon.

### Weaning

The mean age at weaning among the participants was 7.4 ± 3 months.

**Table 3:** Socioeconomic and environmental risk factors for malnutrition

	Case	Control	Odds Ratio OR	95% Confidence Interval CI	*P* value
**Gender**			1.45	0.907–2.325	0.060
Male	79	66			
Female	61	74			
**Monthly household income**			3.164	1.41–7.06	0.002
< 25,000 PKR (<$84)	131	115			
≥ 25,000 PKR (≥$84)	9	25			
**Migration**					
Settled in Karachi since < 15years	93	80	1.484	0.9–2.41	0.055
Settled in Karachi ≥ 15years	47	60			
**Mother’s Educational level**			1.84	1.1–2.97	0.0066
Illiterate	91	70			
Primary and above	49	70			
**Father’s Educational level**	**Case**	**Control**	2.56	1.47–4.4	0.0004
Illiterate/Primary	115	90			
Secondary and above	25	50			
**Mother’s Working status**	**Case**	**Control**	1.2	0.6–2.4	0.298
Working	20	17			
Housewife	120	123			
**Father’s Employment status**	**Case**	**Control**	0.79	0.2-3.0	0.368
Jobless	4	5			
Employed	136	135			
**Father’s job type**			1.78	0.1–2.86	0.0086
Daily Wage	78	58			
Temporary stable/ Permanent	62	82			
**House hold Type**			1.03	0.6–1.6	0.45
Nuclear family	48	47			
Joint family	92	93			
**Number of siblings**			1.68	0.99–2.8	0.025
3 or more	106	91			
2 or less	34	49			
**No. of rooms**			2.1	1.13–4.1	0.02
2 or less	123	108			
3 or more	17	32			
**Type of toilet**			4.46	2.7–7.34	0.0001
Pour/Pit latrine/open sewer	96	46			
Flush/VIP latrine	44	94			

There was no significant association between malnutrition and having siblings under the age of 2 years (OR = 1.22, *p* value = 0.4). There was no association between living in a rented house and malnutrition (OR = 0.73, *p* value 0.2).

Most of the participants were weaned after 6.5 months (*n* = 154;55%). Only 88 (31.4%) mothers weaned their children at 6 months. Inappropriate weaning age was observed in 192 (68.5%) participants, a majority of whom were cases, as shown in Table 4.

**Table 4:** Child related risk factors for malnutrition

Risk factor	Case	Control	Odds ratio OR	95%confidence interval	*P* value
**Delivery place**			1.87	1.14–3.08	0.007
Home/private clinic	100	80			
Hospital	40	60			
Total	140	140			
**Delivery conducted by**			1.91	1.18–3.08	0.004
Dai/Relative/LHW	91	69			
Doctor/Trained Midwife	49	71			
**Birth weight**			1.17	0.6–2.22	0.625
LBW	24	21			
Normal weight	116	119			
Total	140	140			
**Immunization**			1.56	0.889–2.73	0.060
Incomplete for age/no record	38	27			
Complete for age	102	113			
**Breast feeding**			3.778	2.22–6.4	0.0001
No Exclusive for 6 months	112	72			
Exclusive breastfeeding for 6 months	28	68			
**Weaning age**			2.593	1.53–4.39	0.0002
Early or late (< 6 or > 6.5 months)	110	82			
Appropriate (6-6.5 months)	30	58			
**Frequency of common illnesses**			1.02	0.6–1.6	0.453
Once in 2 months or more frequently	70	69			
Once in 3 months or occasionally	70	71			
**History of worm infestation**			1.35	0.7–2.5	0.171
Yes	27	21			
No	113	119			
**Soil/mud eating habit**			0.56	0.2–1.4	0.120
Yes	7	12			
No	133	128			

The significant results of the multivariate analyses are presented in Table 5. After adjusting for the age of the child, source of water, presence of a cemented or uncemented house, number of people using one toilet and all other factors in the model.

**Table 5:** Significant results of multinomial logistic regression

Risk factor	Adjusted odds ratio	95% confidence interval	*P*-value
Father’s education primary or less	4.86	2.23–10.60	0.0001
Monthly income less than 25 thousand PKR ($89) a month	7.134	1.67–30.54	0.008
Pour/Pit latrine/Open sewer type of toilet	4.41	2.67–7.3	0.0001
Inappropriate duration of exclusive breast feeding (< 6 months)	3.578	1.58–8.08	0.002
Inappropriate weaning age (Early or Delayed)	3.71	1.53-9.0	0.004

## Discussion

Malnutrition is a multifactorial illness and children can be affected by a wide variety of modifiable and non-modifiable determinants. This study endorsed various risk factors that were consistent with the findings of previous studies and the risk of some factors was greater than that of others. The association between economic status and malnutrition is well documented [16]. This study showed similar findings. Although the community under study is overall a poor community, the malnutrition risk of households with low family income earning less than or equal to national minimum wage for unskilled work in Pakistan, i.e., PKR 25,000/= was very high after adjustment (aOR = 7.1). This reiterates that the role of economics in determining health can never be over emphasized.

The majority of the cases were males, and the majority of controls were females, similar to the findings by NNS 2018, which found higher percentage of malnourished children under five years of age to be males [4]. However, the association was not significant in our study (OR = 1.45, *p* value = 0.06). The educational status of parents is associated with the nutritional status of the child. Most previous studies have highlighted mothers’ education as an important factor [17–19]. In this study paternal education (primary or below) was found to be more strongly associated with malnutrition than low maternal education. A possible explanation could be that fathers play a leading role in patriarchal tribal communities even if they migrate and reside in urban squatters. Daily wagers are vulnerable in many ways and this was reflected in the current study, showing a daily wager father is 1.8 times more likely to be existing among cases (*p* value = 0.008).

Although most of the participants were born in Karachi, their parents had moved to the city from other parts of Pakistan or from Afghanistan less than fifteen years ago (61.8%). The risk of malnutrition was 1.48 times greater for children whose parents had migrated less than fifteen years ago. However, the result was not significant (*p* value 0.055). Reporting bias is very likely as many participants are suspected to have hidden their migration history from Afghanistan due to current sociopolitical issues, including the risk of deportation. Since most Afghans are ethnically Pashtoon, it is sometimes difficult for a non-Pashtun to differentiate between a Pakistani Pashtun and an Afghan Pashtun.

Environmental factors are strong predictors of malnutrition [20, 21]. Some past studies point to crowding as a predictor of malnutrition [22]. In the current study, the number of rooms in the house being two or less was independently associated with undernutrition (OR = 2.1). The cases and controls were almost equally divided into nuclear and joint families. The majority of both cases and controls lived in joint families and no association was found with household type (OR = 1.03). The cases were much more likely to have three or more siblings (OR = 1.68). A large family size may be associated with poor care. A greater number of children is usually associated with other socioeconomic factors such as parents’ education and income and this finding was not significant after adjustment.

Six months exclusive breastfeeding of infants is recommended for achieving optimal growth and physical and mental development [23]. Breastfeeding should be continued for up to two years, along with initiation of weaning at six months. In this study, only 38.5% of the children were exclusively breastfed for 6 months [21]. Pakistan is the country with lowest exclusive breastfeeding rates in South Asia [24]. It has been observed that bottle feeding is widespread in poor households and higher than recommended dilutions are used by parents due to affordability issues. A low concentration of formula further deteriorates nutrition, on top of other bottle associated risks. The present study showed that not only formula but also buffalo and goat milk as well as tea whitener were given to infants. A staggering 6% of the children (mostly cases) were given tea whiteners instead of breastmilk or even formula. This is a very serious issue that needs to be addressed. Parents must be educated about the serious impact of such use. In the present study, the inappropriate duration of exclusive breastfeeding was associated with 3.58 times risk of malnutrition after adjustment. Breastfeeding promotion is thus an urgent and efficient intervention to fight malnutrition in the children under five years of age.

Weaning should be timely, adequate, safe and proper [25]. Majority of the participants (68.5%) showed inappropriate weaning age, mostly delayed. Only 31% participants were weaned at the WHO recommended age of six months. The association with malnutrition was strong, and inappropriate weaning age was associated with more than three times malnutrition chance in the present study (aOR = 3.7).

Several studies have shown an independent association between the incidence of common illnesses such as diarrhea, cough and cold and the development of malnutrition. Acute infections can lead to weight loss, and being underweight can suppress immunity increasing risk of infections. Thus, a vicious cycle occurs [15, 21]. However, the current study failed to establish an association between frequent common illnesses and malnutrition.

The current study did not find any strong association with low birth weight (OR = 1.17). This finding contrasts with the previous studies [15, 21, 26]. However, a possible explanation is that most of the participants were born at home or so-called private clinics run by nontrained staff, and there was no documented evidence of birth weight. The mothers’ recall of weight could thus not be trusted. This study revealed an association between delivery at home/private clinic and malnutrition (OR = 1.87), but this finding lost significance after adjustment.

An association between a birth attendant being a relative or traditional attendant (Dai) and malnutrition was also established in the present study (OR = 1.91). Though, the adjusted rates did not verify this association perhaps because home deliveries by nontrained staff could be a result of various socioeconomic determinants that are independent risk factors for malnutrition, such as maternal education, low income, lack of assimilation in urban society, distrust of healthcare facilities and/or poor access to healthcare. This phenomenon needs to be explored.

This study showed significant association between pour/pit/open sewer and malnutrition, which was consistent after adjustment (aOR = 4.41). This study showed that environmental factors such as toilet facilities are important predictors. Children with access to VIPs or flush toilets had a decreased risk of malnutrition. Unhygienic conditions such as open defecation and pour or pit latrine are associated with malnutrition according to many past studies conducted in rural settings [27]. These findings are consistent with the findings of the present study, which showed four times higher risk of malnutrition in children using pour pit toilets.

Children with complete Immunization status did not have a lower risk of malnutrition, unlike in some previous studies on the subject [28, 29] The children showed overall better immunization coverage (*n* = 215) than the national coverage, probably due to urban setting of a primary healthcare center providing immunization facilities.

One limitation of the current study is that it employed consecutive sampling technique in a primary healthcare setting. Further community bast studies with probability sampling and better generalizability are needed for a clearer picture.

A very disturbing experience while conducting the current study was that it was easier and quicker to enroll cases while it became very difficult to find controls who met the inclusion criteria. It took us extra months to find children who did not have z scores below − 2 SD of the WHO cut-off for WFA, HFA, WFH and OFC. It is therefore suspected that majority (> 50%) of the children under five years of age in the community are malnourished. A screening survey in the community could give a broad picture of the burden of malnutrition in the urban squatters of Karachi, that may well be above the national or district- level average. A very high prevalence of malnutrition is suspected in the community and a prevalence study in the same community is needed to highlight it.

## Conclusion

Low family income, low paternal education, poor toilet facilities, lack of exclusive breastfeeding for first six months of life and inappropriate weaning age, were significant predictors of childhood malnutrition. Some variables which were found to be significant at univariate analyses were mother’s education, a daily wager father, number of siblings ≥ 3, number of rooms ≤ 2, and home delivery of a child or delivery by a nonskilled birth attendant, such as a child’s relative/Dai/LHV.

The implementation of poverty reduction programs and sanitation provision at affordable rates is needed for health improvement. The economic conditions of Pakistan are deteriorating, and improving the quality of life for urban slum residents may be long term and ambitious, but simple practical measures, such as exclusive breast feeding for six months, and appropriately timed weaning, are cost effective methods that can improve the nutritional status without financial burdens on parents. The parents need to be educated on this subject. Implementing strategies to create supportive environments for enabling underprivileged mothers to successfully employ best breastfeeding practices is urgently needed and should be given priority by stake holders. The nutritional status of urban squatter settlement children is very poor and needs special consideration.

## Data Availability

The data are from vulnerable and conservative tribal communities residing in urban squatters. Keeping in mind the vulnerability of the population, anonymized data will be shared after approval of the IRB committee of Dow University of Health Sciences if data requestors provide a methodically sound proposal. The corresponding author may be contacted for additional details.

## Declarations

### Principal author’s information

Dr. Gati Ara is an MBBS doctor who acquired her FCPS in Community Medicine. She is currently working as an Assistant Professor in the Department of Community Medicine at Dow Medical College, Dow University of Health Sciences. Her interests are Maternal, Neonatal and Child health (MNCH), Medical Education and Family practice.

## Abbreviations

WFA: Weight-for-age
HFA: Height-for-age
WFH: Weight-for-height
OFC: Occipitofrontal circumference
EPI: Expanded Program on Immunization
LHV: Lady Health Visitor
